# Harpin-induced expression and transgenic overexpression of the phloem protein gene *AtPP2-A1 *in Arabidopsis repress phloem feeding of the green peach aphid *Myzus persicae*

**DOI:** 10.1186/1471-2229-11-11

**Published:** 2011-01-13

**Authors:** Chunling Zhang, Haojie Shi, Lei Chen, Xiaomeng Wang, Beibei Lü, Shuping Zhang, Yuan Liang, Ruoxue Liu, Jun Qian, Weiwei Sun, Zhenzhen You, Hansong Dong

**Affiliations:** 1 Key Laboratory of Monitoring and Management of Crop Pathogens and Insect Pests, Ministry of Agriculture of R. P. China, Nanjing Agricultural University, Nanjing, 210095, PR China; 2 Institute of Utilization of Nuclear Techniques, Zhejiang Academy of Agricultural Sciences, Hangzhou, 310021, PR China

## Abstract

**Background:**

Treatment of plants with HrpN_Ea_, a protein of harpin group produced by Gram-negative plant pathogenic bacteria, induces plant resistance to insect herbivores, including the green peach aphid *Myzus persicae*, a generalist phloem-feeding insect. Under attacks by phloem-feeding insects, plants defend themselves using the phloem-based defense mechanism, which is supposed to involve the phloem protein 2 (PP2), one of the most abundant proteins in the phloem sap. The purpose of this study was to obtain genetic evidence for the function of the *Arabidopsis thaliana *(Arabidopsis) PP2-encoding gene *AtPP2-A1 *in resistance to *M. persicae *when the plant was treated with HrpN_Ea _and after the plant was transformed with *AtPP2-A1*.

**Results:**

The electrical penetration graph technique was used to visualize the phloem-feeding activities of apterous agamic *M. persicae *females on leaves of Arabidopsis plants treated with HrpN_Ea _and an inactive protein control, respectively. A repression of phloem feeding was induced by HrpN_Ea _in wild-type (WT) Arabidopsis but not in *atpp2-a1*/E/142, the plant mutant that had a defect in the *AtPP2-A1 *gene, the most HrpN_Ea_-responsive of 30 *AtPP2 *genes. In WT rather than *atpp2-a1*/E/142, the deterrent effect of HrpN_Ea _treatment on the phloem-feeding activity accompanied an enhancement of *AtPP2-A1 *expression. In PP2OETAt (*AtPP2-A1*-overexpression transgenic *Arabidopsis thaliana*) plants, abundant amounts of the *AtPP2-A1 *gene transcript were detected in different organs, including leaves, stems, calyces, and petals. All these organs had a deterrent effect on the phloem-feeding activity compared with the same organs of the transgenic control plant. When a large-scale aphid population was monitored for 24 hours, there was a significant decrease in the number of aphids that colonized leaves of HrpN_Ea_-treated WT and PP2OETAt plants, respectively, compared with control plants.

**Conclusions:**

The repression in phloem-feeding activities of *M. persicae *as a result of *AtPP2-A1 *overexpression, and as a deterrent effect of HrpN_Ea _treatment in WT Arabidopsis rather than the *atpp2-a1*/E/142 mutant suggest that *AtPP2-A1 *plays a role in plant resistance to the insect, particularly at the phloem-feeding stage. The accompanied change of aphid population in leaf colonies suggests that the function of *AtPP2-A1 *is related to colonization of the plant.

## Background

Harpins are multifunctional proteins produced by Gram-negative plant pathogenic bacteria [1,2]. The first-characterized [1] and well-studied harpin [2-7], HrpN_Ea_, is secreted by *Erwinia amylovora*, the bacterial pathogen that causes fire blight disease in rosaceous plants [1]. Multiple functions of harpin proteins, especially in eliciting plant defense responses, were also elucidated initially by studies using HrpN_Ea _as a paradigm [1-3]. Early studies demonstrated that the external application of HrpN_Ea _was able to induce resistance in a variety of plant species [3-7], and that the induced resistance effectively protected plants from attacks by insect herbivores [2,7-9]. HrpN_Ea_-induced resistance to insects first was suggested based on observations of field-grown peppers. Plants that had been treated with HrpN_Ea _incurred fewer injuries from the European corn borer than comparable untreated plants [2]. A deterrent effect on striped cucumber beetles was observed in HrpN_Ea_-treated cucumber; striped cucumber beetles preferred to colonize untreated control plants rather than HrpN_Ea_-treated plants [8]. HrpN_Ea_-induced resistance was also effective in impeding infestations of aphids, an important type of phloem-feeding herbivores [9,10]. In cucumbers grown under environmentally controlled conditions, HrpN_Ea _treatment had a deterrent effect on colonization by the muskmelon aphid *Aphis gossypii *(Glover), which preferred to colonize control plants rather than HrpN_Ea_-treated plants [9]. In *Arabidopsis thaliana *(Arabidopsis), moreover, HrpN_Ea_-induced resistance was shown to repress infestation of the green peach aphid *Myzus persicae *(Sulzer), a generalist phloem-feeding insect [10].

Phloem-feeding insects are highly specialized in their mode of feeding [11] and present a unique stress on plant fitness [12-15]. These insects use their slender stylets to feed from a single-cell type, the phloem sieve element [8,16]. The feeding process can be monitored by the electrical penetration graph (EPG) technique [16]. Pivotally, a stylet puncturing of the host plant cell, shown as a probe in the EPG, may lead to uptake of the phloem sap. In order to prevent protein clogging inside the sieve element, ejection of watery saliva is essential in feeding from the phloem [13,16]. This ejection is detected in the EPG as E1 salivation and always precedes phloem sap ingestion [16]. During ingestion from the sieve element, the watery E2 salivation occurs, and this E2 saliva is added to the ingested sap, thought to prevent phloem proteins from clogging inside the capillary food canal [16]. Therefore, salivation is a crucial event during the phloem-feeding process for insects to overcome a number of phloem-related plant properties and reactions [13-19].

In response to the phloem-feeding stress, plants defend themselves specifically using the phloem-based defense (PBD) mechanism [14-16], which can be also activated by other cues, such as wounding [20-22], besides insect attacks [14,20-22]. Proposed components of PBD include the phloem protein 1 (PP1) and phloem protein 2 (PP2), which represent a type of the most abundant proteins in the phloem sap [23]. PP2 is a phloem lectin conserved in plants [23,24] and is believed to play a role in the establishment of PBD induced by insect attacks [21,25,26] and other stresses, such as wounding [16,21,22,26] and oxidative conditions [25]. In pumpkin, PP1 monomers and PP2 dimers are covalently cross-linked via disulphide bonds, forming high molecular weight polymers that close the sieve pores [21,25,26]. This response is induced by oxidative stress [25] but normally accompanies the synthesis of the β-1,3-glucan callose by callose synthase [20] that accumulates on sieve plates after different stress treatments [21]. Phloem protein plugging and callose closure of sieve pores, and callose coagulation on sieve plates as well, is hypothesized to serve as a physical barrier to prevent the insect from phloem-feeding activity [26]. Nevertheless, evidence for the function of phloem proteins in insect defense has been in paucity.

In the completely sequenced Arabidopsis genome, *PP2 *(previously *PP2*-*like*) genes were identified as a large multigene family constituted of 30 members [23,27], *AtPP2-A1 *to *AtPP2A-15 *and *AtPP2-B1 to AtPP2-B15 *[23]. To our knowledge, however, little has been known about bioprocesses affected by these genes and properties of the encoded proteins. Although Arabidopsis mutants that represent multiple mutation alleles of *AtPP2 *have been generated [27,28], subsequent biological effects have not been studied, and especially, effects of *AtPP2 *mutations on the plant resistance to insects are unclear. For example, different types of Arabidopsis mutants were generated by T-DNA insertion at distinct locations in the *AtPP2-A1 *DNA sequence; *atpp2-a1*/P/-210 resulted from the insertion at nucleotide residue -210 in the promoter region. When grown on an artificial medium, the *atpp2-a1*/P/-210 mutant performs as the wild-type (WT) plant in response to infestations of *M. persicae *adults and newborn nymphs in 24 hours after colonization by the adults [29]. There is as yet no evidence to show if *atpp2-a1*/P/-210 impacts longer behaviors and feeding activities of the insect and if other mutation alleles of *AtPP2-A1 *have biological effects [27,28].

The purpose of this study was to obtain genetic evidence that could elucidate a function of *AtPP2-A1 *in Arabidopsis resistance to *M. persicae*. We began with determining the effect of *AtPP2-A1 *on phloem feeding of aphids that colonized the plants treated with HrpN_Ea _according to previous evidence that the HrpN_Ea _treatment and *M. persicae *infestation had some degrees of overlapping effects on the induction of plant responses. For example, formation of the PP2-PP1 complex needs reactive oxygen burst in cucurbit [25] while reactive oxygen burst is a conserved response in Arabidopsis treated with any harpins [30,31]. *M. persicae *infestation induces an elevation of the ethylene level [32] and triggers modest induction of ethylene-dependent responses [32,33], whereas, HrpN_Ea _induces resistance to *M. persicae *by activating the ethylene-signaling pathway [4,34]. Therefore, we devised to determine the possibility that HrpN_Ea_-induced resistance involves the PBD mechanism to encounter with *M. persicae *infestation. In order to further test this hypothesis, we generated *AtPP2-A1*-overexpression plants and investigated them to elucidate the supposed function of *AtPP2-A1*. In this article, we report evidence that harpin-induced expression and transgenic overexpression of *AtPP2-A1 *induce a repression in the phloem-feeding activity of *M. persicae*.

## Results

### HrpN_Ea _treatment in Arabidopsis induces a repression in phloem feeding and colonization by *M. persicae*

The HrpN_Ea _protein used in this study was produced by prokaryotic expression with a vector that carried a *hrpN*_*Ea *_gene insert; the *hrpN*_*Ea*_-absent Empty Vector Preparation (EVP) that contained inactive proteins but not HrpN_Ea _was used as a control [6]. We investigated activities of *M. persicae *feeding from Arabidopsis (ecotype Col-0) WT plants following treatment with EVP and HrpN_Ea_, respectively. Because a period of five days is usually required for the induction of plant defense responses [3-8], plants at the fifth day posttreatment (dpt) were artificially colonized with uniform ten-day-old apterous (wingless) agamic *M. persicae *females transferred from an Arabidopsis nursery. Aphid feeding activities were studied by the EPG technique applied to 20 aphids that colonized leaves of Arabidopsis plants treated with EVP and HrpN_Ea_, respectively. Feeding activities were depicted as different waveform patterns recognized according to the standard previously established [35] and widely used [13,16,17,36]. Based on the EPG patterns, all the 20 aphids tested in five repetitions of the experiments for each treatment accomplished major steps of the feeding process, but aphid activities varied greatly depending on feeding stages (Table 1).

**Table 1:** Four-hour electrical penetration graph (EPG) analyses of the green peach aphid *Myzus persicae *feeding from wild-type (WT) Arabidopsis plants

Activity examined		Control group mean (SD*)	**HrpN**_**Ea **_treatment group mean (SD*)	Student's *t*-test (n = 20)
Number of nonpuncturing phase	total	13.5 (2.2)	16.0 (3.5)	**
	
	1st h	6.0 (1.0)	13 (2.5)	*p *< 0.01
	
	2nd h	0	0	
	
	3rd h	6.5 (0.8)	2 (0.5)	*p *< 0.01
	
	4th h	1.0 (0.3)	1.0 (0.3)	**

Duration of nonpuncturing, min	total	19.8 (5.2)	16.8 (4.6)	**
	
	1st h	4.9 (0.3)	15.0 (3.9)	*p *< 0.01
	
	2nd h	0	0	
	
	3rd h	11.1 (3.6)	1.3 (0.4)	*p *< 0.01
	
	4th h	3.8 (1.2)	0.5 (0.2)	*p *< 0.01

Time to 1st cell puncturing, min		2.1 (0.6)	2.1 (0.3)	**

Time to 1st pathway, min		3.3 (0.5)	3.0 (0.4)	**

Number of pathway phase	total	19.5 (2.0)	16.5 (1.5)	**
	
	1st h	5.2 (0.5)	11.5 (1.0)	*p *< 0.01
	
	2nd h	3.0 (0.3)	2.0 (0.1)	**
	
	3rd h	7.3 (1.0)	2.0 (0)	*p *< 0.01
	
	4th h	4.0 (0.5)	2.0 (0.2)	*p *< 0.01

Duration of pathway phase, min	total	175.7 (48.9)	205.0 (62.5)	*p *< 0.01
	
	1st h	55.1 (6.7)	45.0 (7.5)	*p *< 0.01
	
	2nd h	37.2 (3.5)	43.9 (7.2)	*p *< 0.05
	
	3rd h	47.4 (5.6)	56.6 (8.0)	*p *< 0.01
	
	4th h	36/0 (3.2)	59.5 (10.5)	*p *< 0.01

Time to 1st phloem phase, min		85.6 (10.7)	104.3 (12.0)	*p *< 0.01

Number of cell puncturing after 1st phloem phase		20.5 (2.0)	11 (1.6)	*p *< 0.01

Number of phloem phase	total	7 (1.0)	3.0 (0.2)	*p *< 0.01
	
	1st h	0	0	
	
	2nd h	3.0 (0.5)	1.5 (0.5)	*p *< 0.01
	
	3rd h	1.0 (0)	1.5 (0.5)	**
	
	4th h	3.0 (0.5)	0	*p *< 0.01

Duration of phloem phase, min	total	44.5 (8.5)	18.2 (3.6)	*p *< 0.01
	
	1st h	0	0	
	
	2nd h	22.8 (5.0)	16.1 (3.5)	*p *< 0.01
	
	3rd h	1.5 (0.5)	2.1 (0.6)	*p *< 0.01
	
	4th h	20.2 (3.5)	0	*p *< 0.01

Duration of phloem feeding, min	total	44.5 (8.5)	18.2 (3.6)	*p *< 0.01
	
	E1	12.6 (2.8)	5.0 (1.4)	*p *< 0.01
	
	E2	31.9 (3.5)	13.2 (3.1)	*p *< 0.01

*SD, standard deviation. **Insignificant difference at *p *< 0.05.

**Figure 1:**
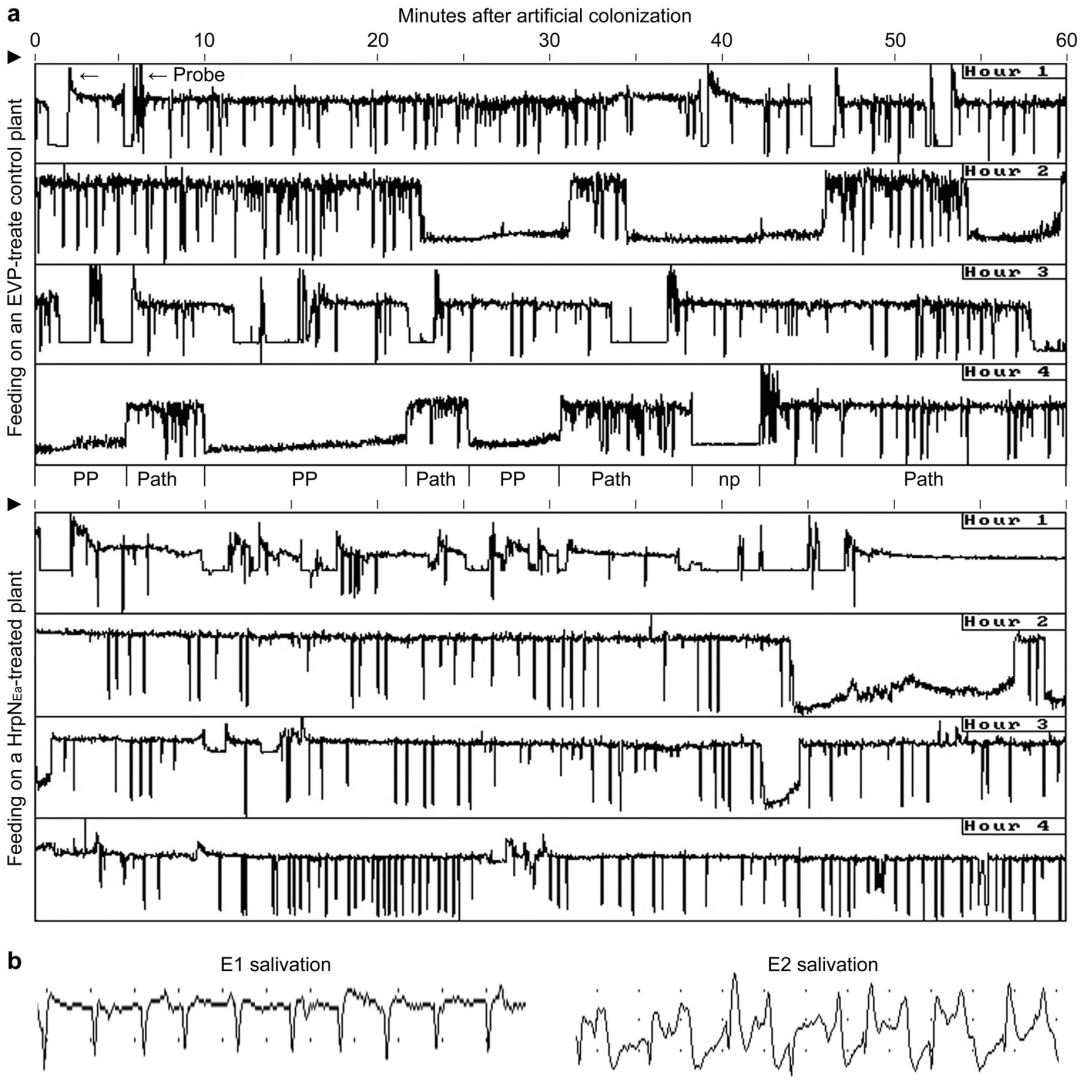
**PG patterns and waveforms of the green peach aphid *Myzus persicae *on wild-type (WT) Arabidopsis**. **(a) **Four-hour EPG record. Plants were treated with the bacterial harpin protein HrpN_Ea _and specific control protein preparation EVP, respectively. Five days later, uniform ten-day-old apterous aphid females were placed on upper sides of the top first expanded leaves. Feeding activities were detected immediately with a four-channel Giga-4 direct current amplifier, which enabled simultaneous recording from four individual aphids. The EPG record represents 20 aphids feeding on 20 plants treated differently and monitored in five repetitions of experiments. Reiteratively appeared EPG waveforms are indicated once at proper spaces. PP, phloem phase; Path, pathway phase; np, no probing. **(b) **Two important waveforms in the phloem phase dissected every five second using the EPG analysis software STYLET 2.5.

Figure 1a shows a four-hour EPG record of aphid feeding from the WT plant. The nonpuncturing phase (Figure 1a, np) indicated the stylet staying outside the cuticle. Cell puncturing (Figure 1a, probe) led to the pathway phase (Figure 1a, path) in which the stylet penetrated between cells en route to the vascular tissue [35]. In the four-hour EPG record, total number and duration of the nonpuncturing phase, time to the first cell puncturing or the first pathway phase, and total number and duration of the pathway phase were all similar in HrpN_Ea_-treated plants as in control plants (Table 1). The pathway phase represents insect's efforts in navigating the phloem and preparing to ingest sap from sieve elements [16,17]. Subsequently, aphids may proceed to the phloem phase (Figure 1a, PP) in which ingestion of the phloem sap may occur [16]. The pathway phase may be also connected with the xylem phase, indicating stylet penetration of the xylem in the vascular tissue [16], but xylem phase was not found in this study. Analyses of the four-hour EPG record as a whole suggested that the plant treatment with HrpN_Ea _did not evidently change aphid activities outside vascular tissues when evaluated in a four-hour course of surveys (Table 1). However, analyses by hour offered additional information. In the first hour, especially, the nonpuncturing phase was more frequent with longer duration while the pathway phase was more but shorter under the HrpN_Ea _treatment condition compared with control. This result suggested that the HrpN_Ea _treatment impeded aphids in early feeding activities, both puncturing of the plant cell and navigating of the phloem. Subsequently, however, the phloem phase was always shorter, in HrpN_Ea_-treated plants than in control plants, no matter if the EPG patterns were analyzed by hour or based on the four-hour record as a whole (Table 1).

Based on the four-hour EPG record, the proportions of times within the pathway phase and time to the first phloem phase were much longer, suggesting the impediment to aphids in locating the ingestion site within the vascular tissue, in HrpN_Ea_-treated plants compared with control plants (Figure 1a; Table 1). On HrpN_Ea_-treated plants, moreover, aphids took fewer actions to puncture cells (Table 1, Number of cell puncturing) and to enter the phloem phase (Table 1, Number of phloem phase) after the first entry of phloem phase. These results suggested that phloem properties of HrpN_Ea_-treated plants were changed as unfavourable to aphid feeding. In consistence with this notion, total duration of the phloem phase was markedly shorter in HrpN_Ea_-treated plants than in control plants (Table 1). Noticeably, duration of the phloem phase in the second hour of the EPG monitoring, being 30 in HrpN_Ea_-treated plants and 14 min in control plants, on average, strongly suggested the deterrent effect of the HrpN_Ea _treatment on the phloem-feeding activity of *M. persicae*.

In the phloem phase, E1 and E2 salivations were recognized by dissecting the EPG waveform patters (Figure 1b). Compounds of E1 and E2 saliva produced by aphids after stylet entry of the phloem are believed to function in preventing protein clogging inside the sieve element and preventing phloem proteins from clogging inside the capillary food canal, respectively [16]. Thus, E1 and E2 salivations play an important role in ingestion of the phloem sap by the insects [13,16]. As shown in Table 1, durations of both E1 and E2 salivations were much shorter in HrpN_Ea_-treated plants than control plants, confirming the deterrent effect of the HrpN_Ea _treatment on the phloem-feeding activity of *M. persicae*.

**Figure 2:**
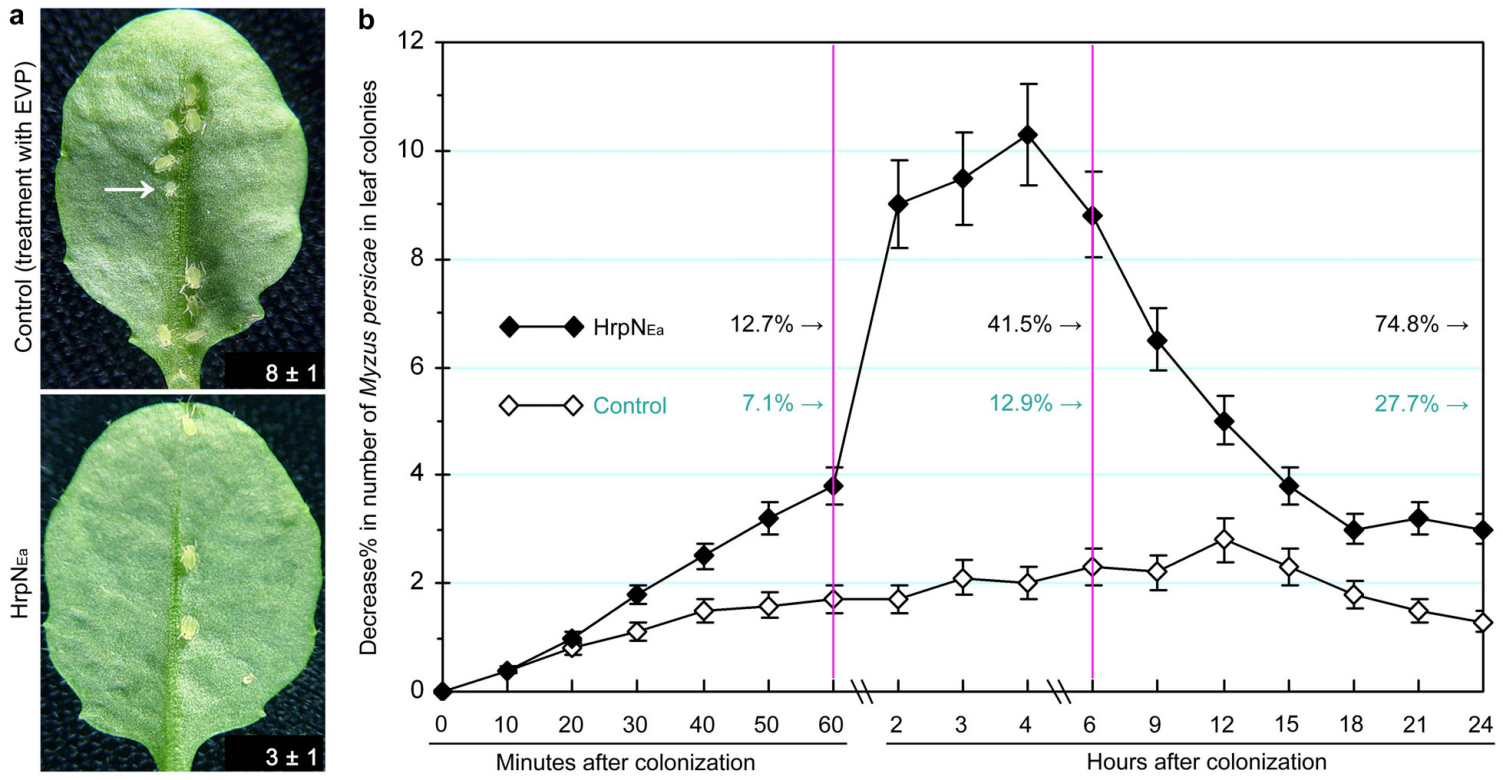
**24-hour monitoring of *M. persicae *population in leaf colonies**. **(a) **Appearance of aphid colonies on leaves. WT plants were treated with HrpN_Ea _and EVP, respectively. Five days later, uniform aphids were placed on lower sides of the top two expanded leaves, 10 aphids/leaf; leaves were photographed 24 hours later. The arrowhead points a nymph produced after leaf colonization. The numerical values, given as mean ± standard deviation (SD), indicate the number of aphids that stayed on the leaf colony for 24 hours. A photo represents 120 leaf colonies on 60 plants. **(b) **Changes of aphid population in 24 hours. Leaf colonies on plants from (a) were surveyed, the number of aphids that stayed in a leaf colony was scored, and percent decrease in the number of aphids that left the leaf colonies was calculated as mean ± standard deviation (SD) of replicate results (n = 120 leaf colonies). The numerical values indicate total proportions (means ± SDs) of decreases in aphid populations within 1, 6 and 24 hpt (hour posttreatment).

To correlate repression in the phloem-feeding activity with colonization of Arabidopsis by *M. persicae*, we monitored a large-scale population of the insect and surveyed a 24-hour fluctuation in leaf colonies. A total of 1,200 uniform individuals of apterous and agamic *M. persicae *females were monitored in four repetitions of the experiments for plants treated with EVP and HrpN_Ea_, respectively. The number of aphids that stayed in their colonies on leaves was counted and the number of aphids that run away from the leaf colonies was calculated at intervals in 24 hours (Figure 2). At each time point, the number of aphid individuals run away from their colonies on leaves of HrpN_Ea_-treated plants was greater than the number of the insect run away from colonies on leaves of control plants (Student's t-test, *P *< 0.01). Proportions of aphids escaped from leaf colonies in control plants were close at the different intervals, but much higher proportions of aphid escapes from leaf colonies in HrpN_Ea_-treated plants were observed in the short period of two to four hours. And this period was critical to the effect of HrpN_Ea _treatment on colonization of the plant, consistent with the effect on the phloem-feeding activity (Figure 1a). In 24 hours, a total of 74.8% aphids on average run away from their colonies on leaves of HrpN_Ea_-treated plants, in contrast to totally 17.7% aphids escaped from leaf colonies in control plants (Figure 2; Student's t-test, *P *< 0.01). In subsequent days, aphids that had run away from the original leaf colonies were found in a drifting status, died, and appeared as white carcases on other different parts of the plants. These observations indicate that the HrpN_Ea _treatment impairs the stability of Arabidopsis colonization by *M. persicae*.

### Arabidopsis *atpp2-a1*/E/142 mutant pampers *M. persicae *in phloem feeding

**Figure 3:**
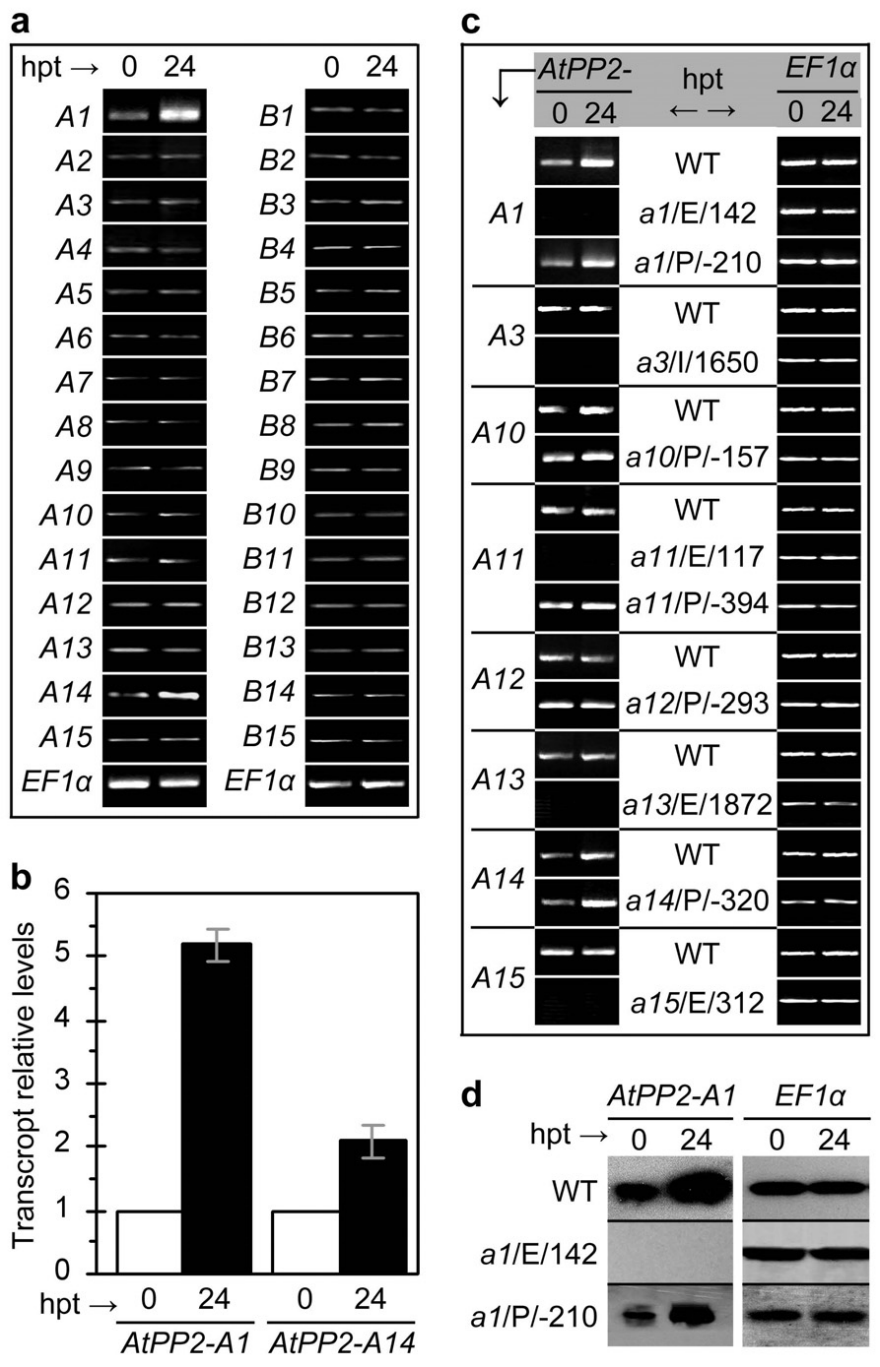
**Analyses of *AtPP2 *gene expression**. **(a-d) **Plants were treated with HrpN_Ea _and sampled at 0 hpt (immediately after treatment) and 24 hpt. Gene expression was determined by Reverse transcriptase-polymerase chain reaction (RT-PCR) using *EF1α *as a reference gene, by real-time RT-PCR using *EF1α *and *ACTIN2 *genes as references, or by northern blot hybridization with specific probes. **(a) **RT-PCR analyses of gene expression in WT plants. *AtPP2-A1 *through *AtPP2-A15 *and *AtPP2-B1 *through *AtPP2-B15 *are abbreviated as *A1 *through *A15 *and *B1 *through *B15*, respectively. **(b) **Real-time RT-PCR analysis of *AtPP2-A1 *and *AtPP2-A14 *expression in WT plants. Gene transcript was quantified as mean ± SD (n = 4 repeats) relative to reference genes and normalized to null-template controls. **(c) **RT-PCR analyses to determine effects of the WT plant and *AtPP2-A*-modified mutants on expression of selected *AtPP2-A *genes. The sequence-indexed T-DNA insertion mutants are shown as ellipsis of prefixal *atpp2-*. **(d) **Northern blots hybridized with probes specific to *AtPP2-A1 *or *EF1α*. Both mutants are shown in abbreviated form.

To gain information about relationships between previously identified 30 *AtPP2 *genes [23] and HrpN_Ea_-induced repression in the phloem-feeding activity of *M. persicae*, we studied expression of these genes in HrpN_Ea_-treated WT Arabidopsis plants. Reverse transcriptase-polymerase chain reaction (RT-PCR) was performed using the *EF1α *gene as a reference [6,37] to detect the expression of 15 *AtPP2-A *genes and 15 *AtPP2-B *genes [23]. As shown in Figure 3a, transcript levels of the genes, except *AtPP2-A1 *and *AtPP2-A14*, in HrpN_Ea_-treated plants were similar when tested at the 24th hour posttreatment (hpt) as tested at 0 hpt (immediately after the plant treatment). However, both *AtPP2-A1 *and *AtPP2-A14 *were expressed at enhanced extents in HrpN_Ea_-treated plants. Subsequent real-time RT-PCR analyses using the *EF1α *and *Actin2 *genes as references [37,38] revealed a greater expression level of *AtPP2-A1 *than *AtPP2-A14*. Relatively, *AtPP2-A1 *and *AtPP2-A14 *transcripts accumulated in 24 hours were 5 and 2 times more, respectively, in HrpN_Ea_-treated plants than in control plants (Figure 3b).

To correlate the role of HrpN_Ea _in enhancing gene expression with the role in repressing phloem feeding of *M. persicae*, we investigated Arabidopsis mutants previously generated by T-DNA insertion at *AtPP2-A *sequences. Two *AtPP2-A1 *sequence-indexed lines were chosen for the test because the AtPP2-A1 protein had been shown to affect weight gain in *M. persicae *nymphs [24], and the other eight *AtPP2-A*-modified mutants were considered for comparison because the *AtPP2-A *genes differed from *AtPP2-A1 *in response to HrpN_Ea _(Figure 3a). The ten mutants were confirmed for the presence of T-DNA insert according to available information (Table 2); they were named conventionally after lowercase gene symbols, suffixed with the insert locations, including gene DNA components (P, promoter; E, exon; I, intron) and nucleotide residue sites at the gene DNA sequences. Mutants were compared with WT in expression of the corresponding genes and aphid behaviors on leaf colonies.

**Table 2:** Information on *AtPP2-A*-defected Arabidopsis mutants investigated in this study

Gene name	Locus no.	Mutant name	T-DNA insertion site	**Mutant seed stock no.**^**a**^	**TAIR**^**b **^**annotations**
*AtPP2-A1*	AT4G19840	*atpp2-a1*/E/142	Exon, 142	CS837256	T-DNA insertion lines; a modified approach of thermal asymmetric interlaced-PCR was used to amplify DNA fragments flanking the T-DNA left border from the transformed lines; no phenotype information available at this time.
	
*AtPP2-A11*	AT1G63090	*atpp2-a1*/P/-394	Promoter, -394	CS842726	

*AtPP2-A1*	AT4G19840	*atpp2-a1*/P/-210	Promoter, -210	SALK_080914C	Sequence-indexed T-DNA insertion lines; presence of the insertion was analyzed by PCR; kanamycin resistance gene may be silenced; PCR- or hybridization-based segregation analysis is required to confirm presence and homozygosity of insertion; may be segregating for phenotypes that are not linked to the insertion; may have additional insertions potentially segregating; no phenotype information available at this time.
	
*AtPP2-A10*	AT1G10155	*atpp2-a10*/P/-157	Promoter, -157	SALK_107807C	
	
*AtPP2-A3*	AT2G26820	*atpp2-a3*/I/1650	Intron, 1650	SALK_005443C	
	
*AtPP2-A11*	AT1G63090	*atpp2-a11*/E/117	Exon, 117	SALK_080546	
	
*AtPP2-A12*	AT1G12710	*atpp2-a12*/P/-293	Promoter, -293	SALK_015774	
	
*AtPP2-A13*	AT3G61060	*atpp2-a13*/E/1872	Exon, 1872	SALK_046907	
	
*AtPP2-A14*	AT5G52120	*atpp2-a14*/P/-320	Promoter, -320	SALK_066553	
	
*AtPP2-A15*	AT3G53000	*atpp2-a1*/E/312	Exon, 312	SALK_022649	

^a ^Distribution seeds of *atpp2-a1*/P/-210, *atpp2-a10*/P/-157 and *atpp2-a3*/I/1650 are from confirmed lines and T2 or T3 generation for the other mutants.

^b ^TAIR, The Arabidopsis Information Resource http://www.arabidopsis.org databases.

Parallel RT-PCR analyses of RNA samples isolated at 0 and 24 hpt revealed that the *AtPP2-A *genes performed differently in corresponding mutants compared with the WT plant (Figure 3c). Both the basal expression (0 hpt) and HrpN_Ea_-induced expression (24 hpt) of *AtPP2-A1 *was detected in the *atpp2-a1*/P/-210 mutant as in WT but not in the *atpp2-a1*/E/142 mutant (Figure 3c). This result was confirmed by northern blot hybridization (Figure 3d). And this result conformed to the PLACE Web Signal Scan [39], which revealed 37 types of *cis*-acting regulatory DNA elements present in the predicted 344-bp promoter of *AtPP2-A1*. Eighteen elements exist as a single copy and 19 elements have multiple copies, located at distant 83 sites in the promoter sequence. However, none of the elements was disrupted by T-DNA insertion and this might account for *AtPP2-A1 *expression in *atpp2-a1*/P/-210. Similarly, none of 35 types of *cis*-acting regulatory DNA elements scanned in the upstream -370 region of the *AtPP2-A14 *DNA sequence was disrupted in *atpp2-a14*/P/-320. This mutant performed as WT in both the basal expression and HrpN_Ea_-induced expression of *AtPP2-A14 *(Figure 3c). The other eight mutants behaved differently in expression of the corresponding *AtPP2-A *genes. *AtPP2-A3*, *-A11*, *-A13*, and *-A15 *were not expressed in their corresponding mutants *atpp2-a3*/I/1650, *-a11*/E/177, *-a13*/E/1872, and *-a15*/E/312. In contrast, *atpp2-a10*/P/-157, *a11*/P/-394, *a12*/P/-293, *a14*/P/-320 performed as WT in the expression of the corresponding *AtPP2-A *genes. In *atpp2-a12*/P/-293 and *atpp2-a12*/P/-293, T-DNA insert did not disrupt any DNA regulatory motifs present in *AtPP2-A11 *and *AtPP2-A12 *promoters. In *atpp2-a10*/P/-157, T-DNA insert disrupted the pollen-specific transcription activator element AGAAA (#S000245) [40,41] located between -159 and -155 in the *AtPP2-A10 *sequence. In *atpp2-a11*/P/-394, the MYB recognition site TGGTTT (#S000408) [42] located between -398 and -393 in the *AtPP2-A11 *sequence was disjoined by T-DNA insertion. However, both mutations did not affect basal expression of the genes (Figure 3c). In the ten mutants, therefore, only *atpp2-a1*/E/142 represents an effective mutation allele, which may be responsible for a transcriptional stop of *AtPP2-A1 *in the plant and result in experimental compromises in both the basal expression and HrpN_Ea_-induced expression of the gene.

**Figure 4:**
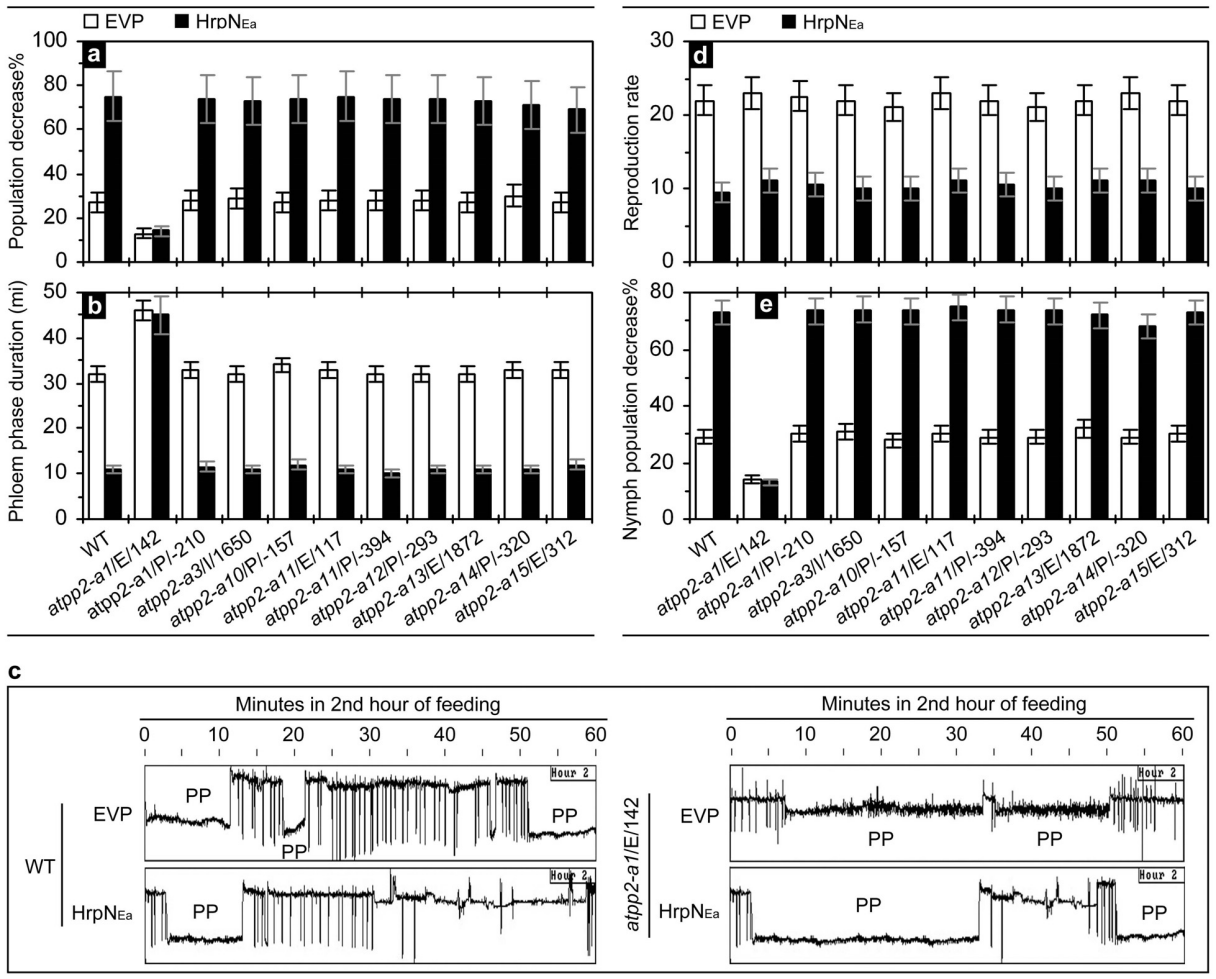
**Comparison of Arabidopsis *AtPP2-A*-modified mutants and WT plant in colonization and phloem feeding by aphids**. **(a) **Changes of aphid population in 24 hours. Plants were treated with HrpN_Ea _and EVP, respectively. Five days later, uniform aphids were placed on lower sides of the top two expanded leaves (10 aphids/leaf). The number of aphids that stayed in a leaf colony was scored at the 24th hour after leaf colonization. Percent decrease (mean ± SD; n = 120 leaf colonies) in the number of aphids that run away from the leaf colonies was calculated. **(b) **Total duration of the phloem phase in a four-hour EPG monitoring course. Plants treated as in (a) were colonized by aphids at the fifth day after treatment; uniform aphids were placed on upper sides of the top first expanded leaves. Feeding activities were detected immediately with a four-channel current amplifier system, and total duration of the phloem phase (mean ± SD; n = 20 aphids) was scored. **(c) **The second-hour EPG record particularly indicating the phloem phase (PP) in WT and an *AtPP2-A1*-defected mutant. Experiments were the same as in (b). The EPG record represents 20 aphids feeding from 20 plants of WT and the mutant, respectively. **(d, e) **Reproduction of aphid adults and colonization behaviors of newborn nymphs. Experiments were similar as in (a) and insects were surveyed in five days after colonization of leaves by adults. Reproduction rate was given as the ratio between total number of newborn nymphs and total number of adults on leaf colonies. The population decrease was based on total number of nymphs and the number of nymphs that run away from the leaf colony. Data represent mean ± SD (n = 120 leaf colonies).

The ten *AtPP2-A*-modified mutants were compared with the WT plant in terms of colonization and feeding by aphids. Based on monitoring of large-scale populations of apterous and agamic *M. persicae *females (1,200 aphids/treatment/plant genotype), the insect colonies on leaves of *atpp2-a1*/E/142 were stable, shown as a smaller rate of the population decrease in 24 hours, than those on WT and the other nine mutants (Figure 4a; *ANOVA *test, *p *< 0.01). In *atpp2-a1*/E/142, the deterrent effect of HrpN_Ea _on colonization by the insect was little, but the effect was evident in the other mutants as in WT (Figure 4a). Based on the four-hour EPG record, total durations of nonpuncturing and pathway phases had little and insignificant differences between WT and *atpp2-a1*/E/142 under the same condition, HrpN_Ea _treatment or control (Table 3). Then, the four-hour EPG record of aphid feeding from leaves was analyzed to particularly calculate total duration of the phloem phase (Figure 4b), which well reflected HrpN_Ea_-induced repression in aphid feeding from the WT phloem (Table 1). Apparently, aphids preferred to feed from *atpp2-a1*/E/142 (Figure 4c). In the mutant, total duration of the phloem phase in 4 hours was much longer than that in the other mutants and WT as well (Figure 4b; Table 3). Both the second and fourth hour of the EPG record indicated significant deterrent effect of the HrpN_Ea _treatment on aphid feeding from the WT phloem (Table 1), but the deterrent effect was lost in *atpp2-a1*/E/142 (Figure 4c; Table 3). Duration of the phloem phase in the second-hour EPG was much shorter in WT plants treated with HrpN_Ea _vs. EVP, but the duration was close in *atpp2-a1*/E/142 in despite of treatments (Figure 4c; Table 3). These results suggest that *atpp2-a1*/E/142 pampers *M. persicae *in phloem feeding and that *AtPP2-A1 *plays a role in HrpN_Ea_-induced repression of the phloem-feeding activity.

**Table 3:** Four-hour EPG analysis of aphid feeding from WT Arabidopsis and the *atpp2-a1*/E/142 mutant

Activity examined	WT group			*atpp2-a1*/E/142 group		
	
	EVP treatment mean (SD)	**HrpN**_**Ea **_**treatment mean (SD)**	Student's *t*-test (n = 20)	EVP treatment mean (SD)	**HrpN**_**Ea **_**treatment (SD)**	Student's *t*-test (n = 20)
Total duration of nonpuncturing, min	21.1 (4.8)	18.9 (3.5)	*p *> 0.05	31.4 (8.3)	28.5 (6.4)	*

Duration of pathway phase, min	175.0 (50.5)	201.5 (58.6)	*p *< 0.05*	160.0 (42.0)	162.5 (45.5)	*

Total duration of phloem phase, min	43.9 (6.3)	19.6 (3.9)	*p *< 0.005*	48.6 (9.2)	49.0 (11.5)	*

*Insignificant difference at *p *< 0.05.

To gain information about the general function of *AtPP2-A1 *in Arabidopsis resistance to *M. persicae*, we compared *atpp2-a1*/E/142 with the other nine mutants and with WT as well in the effects on multiplication of the insect and subsequent nymph activities. The reproduction rate was scored as the ratio between total numbers of newborn nymphs and total numbers of aphid adults that stayed on leaves in five days after colonization. As shown in Figure 4d, reproduction rates were much smaller under the condition of HrpN_Ea _treatment vs. control (Student's *t*-test, *p *< 0.01) irrespective of the plant genotypes, suggesting that HrpN_Ea_-induced repression of *M. persicae *multiplication [4] was not related to the *AtPP2-A1 *gene. The gene, however, showed a repressive effect on plant colonization by newborn nymphs. Nymph colonies were more stable on *atpp2-a1*/E/142 with a smaller proportion of the population decrease than the other mutants or WT (Figure 4e; *ANOVA *test, *p *< 0.01). In *atpp2-a1*/E/142, the deterrent effect of HrpN_Ea _on colonization by nymphs was little, but the effect was evident in the other mutants as in the WT plant (Figure 4e). Evidently, *AtPP2-A1 *does not affect aphid reproduction, but instead, the gene plays a role in repressing plant colonization by nymphs as by adults.

### *AtPP2-A1*-overexpression confers repressed phloem feeding of *M. persicae*

**Figure 5:**
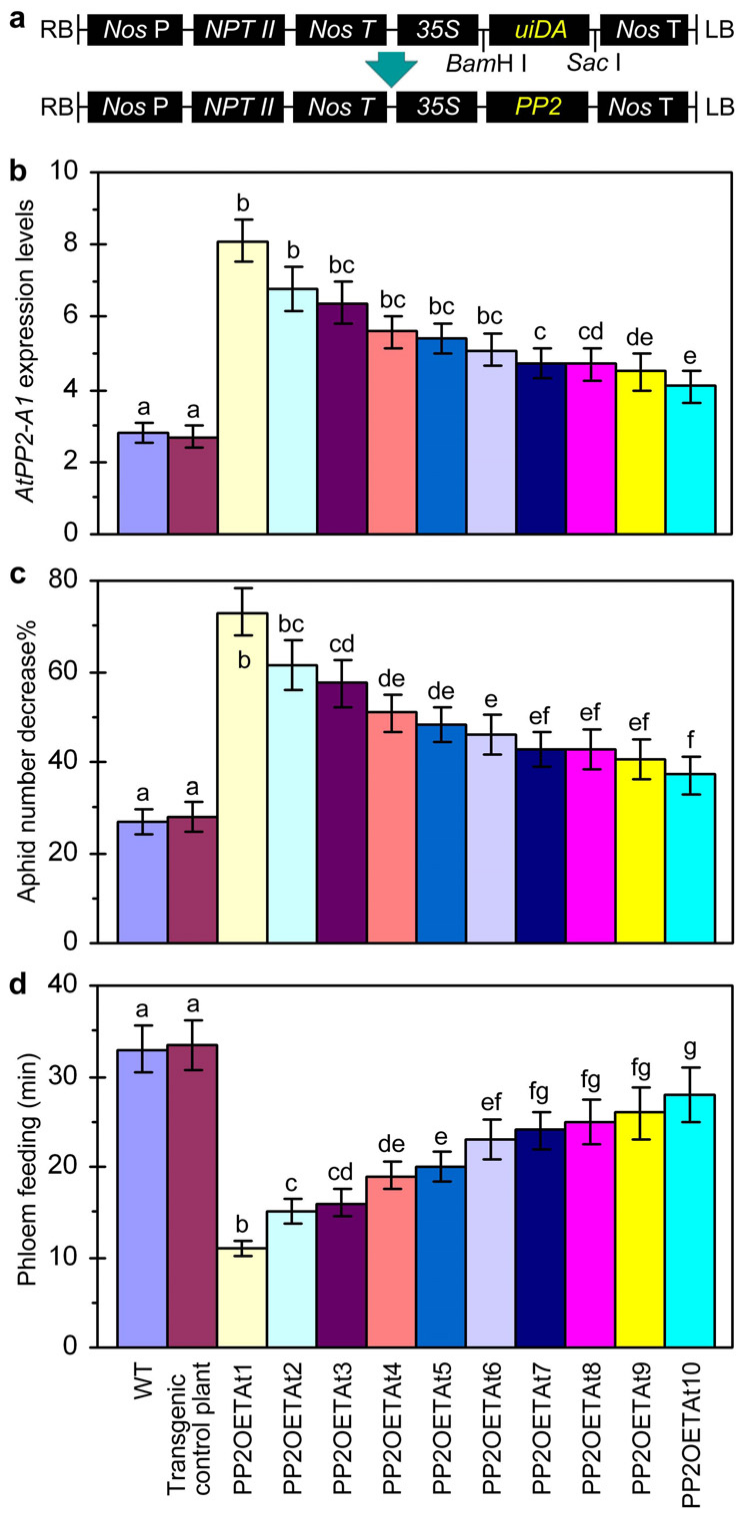
**Genetic construction used in generation of PP2OETAt (*AtPP2-A1*-overexpression transgenic *Arabidopsis thaliana*) and comparison of PP2OETAt and control plants in *AtPP2-A1 *expression and aphid activities on leaves**. **(a) **The construct. The *AtPP2-A1 *(*PP2*) gene was inserted into the binary vector pBI121 at the *Bam*H I and *Sac *I restriction sites to replace *uidA*, a reporter gene encoding β-D-glucuronidase. *Nos *P, promoter from the nopaline synthase-encoding gene (*Nos*); *NPT II*, kanamycin resistance gene; *Nos *T, *Nos *transcription terminator; *35S*, the cauliflower mosaic virus *35S *promoter. **(b-e) **Experiments were done with 35-day-old plants. Different letter labels in histograms indicate significant differences (*ANOVA *test, *p *< 0.01). **(b) **Real-time RT-PCR analysis of *AtPP2-A1 *expression in leaves. The gene transcript was quantified as mean ± SD (n = 3 repeats) relative to reference genes (*EF1α *and *ACTIN2*) and normalized to the null-template control. **(c) **Changes of aphid population in 24 hours. Uniform aphids were placed on lower sides of the top two expanded leaves (10 aphids/leaf). Leaf colonies were surveyed, the number of aphids that stayed in a leaf colony was scored, and percent decrease (mean ± SD; n = 120 leaf colonies) in the number of aphids that run away from the leaf colonies was calculated. **(d) **Total duration of the phloem phase in a four-hour EPG monitoring course. Uniform aphids were placed on upper sides of the top first expanded leaves. Feeding activities were detected immediately with a four-channel current amplifier system, and total duration of the phloem phase (mean ± SD; n = 20 aphids) was scored.

The *AtPP2-A1 *gene was cloned into the binary vector pBI121 under control by the cauliflower mosaic virus 35S promoter (*35S*), creating *pBI121*::*35S*::*AtPP2-A1 *(Figure 5a). Transformation of WT Arabidopsis with the recombinant unit generated PP2OETAt (*AtPP2-A1*-overexpression transgenic *A. thaliana*) plants. Ten PP2OETAt lines were selected and designated as PP2OETAt1 through PP2OETAt10 according to *AtPP2-A1 *expression levels (Figure 5b). Transformation of the WT plant with the empty pBI121 vector, containing neither *uidA *nor *AtPP2-A1*, generated the transgenic control plant, which behaved as WT in all the tests (Figure 5b-5d). Also, WT, transgenic control and PP2OETAt plants did not have evident differences in morphology. Homozygous T3 progenies of the PP2OETAt lines were compared the WT and transgenic control plants in *AtPP2-A1 *expression and in colonization and feeding by apterous *M. persicae *females.

Real-time RT-PCR was conducted with RNA samples from leaves and primers specific to *AtPP2-A1*. As shown in Figure 5b, levels of the *AtPP2-A1 *transcript varied with the different PP2OETAt lines, and levels of the transcript were greater in all the PP2OETAt lines than the transgenic control plant. Compared with the transgenic control plant, PP2OETAt lines seemed more resistant to colonization and feeding by aphids. Smaller populations of aphids were able to stay for 24 hours on leaf colonies of PP2OETAt than the transgenic control plant (Figure 5c). Consistently, aphids preferred to feed from the transgenic control plant rather than PP2OETAt (Figure 5d). Total duration of the phloem phase in the four-hour EPG record was much shorter in PP2OETAt than in the control plant (Figure 5d).

Based on statistical analyses (*ANOVA *test, *p *< 0.01), the ten PP2OETAt lines differed significantly from the transgenic control plant in levels of *AtPP2-A1 *expression (Figure 5b), the number of aphids that were able to stay for 24 hours on leaf colonies (Figure 5c), and durations of the phloem phase (Figure 5d). In the ten PP2OETAt lines, the number of aphids that were able to stay for 24 hours on leaf colonies was increased (Figure 5c), but durations of the phloem phase was decreased (Figure 5d), with increases in levels of *AtPP2-A1 *expression (Figure 5b). The PP2OETAt1 line showed as the greatest expresser of *AtPP2-A1 *and the greatest repressor of colonization and feeding by *M. persicae*. In addition, a greater repression of phloem feeding by aphids was observed in the presence than the absence of HrpN_Ea _treatment (not shown), suggesting that original and introduced versions of the *AtPP2-A1 *gene might be able coordinate their functions and might function simultaneously, in PP2OXTA1.

### *AtPP2-A1 *expression in different organs of PP2OETAt1 is consistent with repression of phloem feeding by *M. persicae*

**Figure 6:**
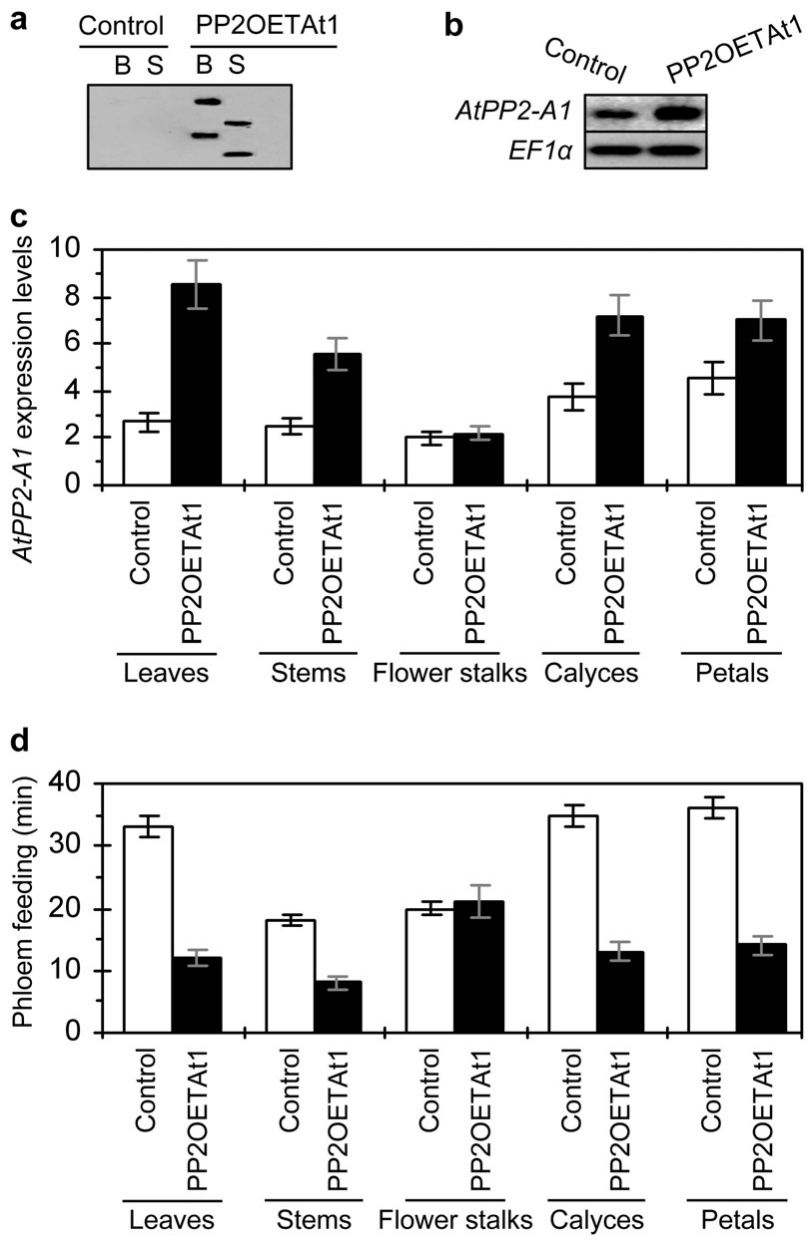
**Comparison of PP2OETAt1 and transgenic control plants in organ-unspecific *AtPP2-A1 *expression and effects on colonization and phloem feeding by aphids**. **(a, b) **In the experiments, PP2OETAt1 was compared with the transgenic control plant (Control); 35-day-old plants grown in long day were investigated. **(a) **Southern blot hybridized with the *AtPP2-A1*-specific probe. Prior to blotting, the genomic DNA had been digested with the restriction enzymes *Bam*H I (B) and *Sac *I (S). **(b) **Northern blots hybridized with probes specific to *AtPP2-A1 *and the reference gene *EF1α*. **(c) **Real-time RT-PCR analysis of *AtPP2-A1 *expression in the different organs of the plants. The gene transcript was quantified as mean ± SD (n = 3 repeats) relative to reference genes and normalized to null-template controls. **(d) **Total duration of the phloem phase in a four-hour EPG monitoring course. Uniform aphids were placed on the indicated organs. Feeding activities were detected immediately with an EPG monitoring system, and total duration of the phloem phase (mean ± SD; n = 20 aphids) was scored.

PP2OETAt1 was further investigated in the genomic integration of the introduced *AtPP2-A1 *gene, organ specificity of the gene expression, and the effect of *M. persicae *feeding from the phloem. The Southern blot of specifically ingested genomic DNA hybridized with the *AtPP2-A1-*specific probe revealed that the introduced *AtPP2-A1 *gene had been integrated into the genome and existed as a double copy in PP2OETAt1 (Figure 6a). Overexpression of the gene was confirmed by northern blot of leaf RNA samples hybridized with the probe specific to *AtPP2-A1 *(Figure 6b).

Real-time RT-PCR analyses revealed that *AtPP2-A1 *expression varied greatly in different organs of PP2OETAt1. The expression of *AtPP2-A1 *was conspicuous in leaves, stems, calyces, and petals but little transcript was detected from flower stalks (Figure 6c). Amounts of the *AtPP2-A1 *transcript were much greater in leaves, stems, calyces, and petals of PP2OETAt1 than the transgenic control plant. However, close amounts of the transcript were detected from flower stalks of both plants. This result suggested the overexpression of *AtPP2-A1 *in all the organs except flower stalks of PP2OETAt1. Levels of the gene overexpression were higher in leaves, calyces, and petals compared with stems (Figure 6c; *ANOVA *test, *p *< 0.01).

The organ-differential levels of *AtPP2-A1 *overexpression were negatively correlated with the extents by which apterous agamic *M. persicae *females fed from the different organs. Based on total duration of the phloem phase in the four-hour EPG record (Figure 6d), aphids preferred to feed from leaves, calyces, and petals, but aphids were also able to feed from stems and flower stalks. However, durations of the phloem phase were much shorter when aphids were feeding from leaves, stems, calyces, and petals of PP2OETAt1 compared with the transgenic control plant (Student's *t*-test, *p *< 0.01), suggesting that the phloem-feeding activity was repressed in the different organs of PP2OETAt1. Inversely, the phloem phase of aphid feeding from the PP2OETAt1 flower stalk lasted as longer as feeding from the same organ of the transgenic control plant (Figure 6d), suggesting that aphids did not have a preference between both plants in feeding from flower stalks.

### Expression of *AtPP2-A1 promoter-GUS *is organ-unspecific

**Figure 7:**
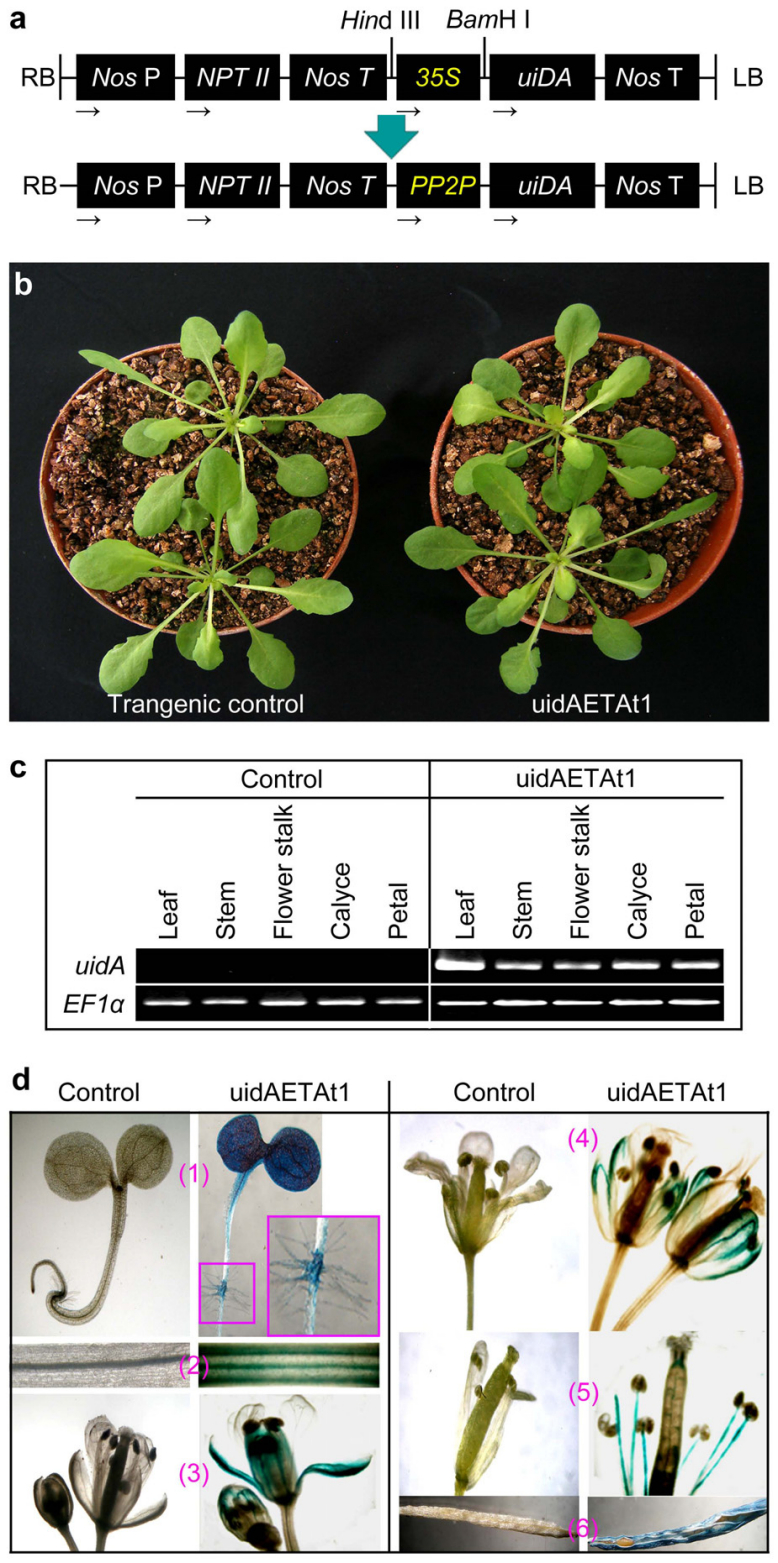
**Genetic construction used in generation of uidAETAt (*AtPP2-A1-promoter-uidA-*expressing *A. thaliana*) and organ-unspecific *uidA *expression in uidAETAt**. **(a) **The construct. The *AtPP2-A1 *promoter (*PP2P*) was inserted into the binary vector pBI121 at the *Hin*d III and *Bam*H I restriction sites to replace *35S *while reserve the reporter gene *uidA *encoding β-D-glucuronidase (GUS). Labels are the same as in Figure 5a. **(b) **Appearance of 35-day-old plants of the uidAETAt1 line compared with the transgenic control plant. **(c, d) **RT-PCR analysis of *uidA *expression and GUS activity in different organs of 35-day-old uidAETAt1 compared with the transgenic control plant.

Because the introduced copies of *AtPP2-A1 *(Figure 6a) are under direction by *35S *(Figure 5a), the organ-differential expression in PP2OETAt1 (Figure 6c) does not offer significant information about organ specificity of the gene expression. Lack of the organ specificity was indicated by the transcript detected from different organs of the transgenic control plant (Figure 6c). In an experimental design to test whether the organ-unspecific *AtPP2-A1 *expression was related with activity of the *AtPP2-A1 *promoter, the promoter placed in front of the *uidA *reporter gene (Figure 7a) was able to drive the gene expression in the uidAETAt (*uidA*-expressing transgenic *A. thaliana*) plant (Figure 7b). Six uidAETAt lines were observed. They seemed to resemble each other closely and were also similar to the transgenic control plant (Figure 7b; uidAETAt1 as a representative line). In the uiDAETAt1 line, *uidA *was expressed markedly in the stem, flower stalk, calyce, and petal, whereas, stronger expression was found in the leaf (Figure 7c). The *uidA *gene encodes β-glucuronidase (GUS) enzyme [43]. GUS activity was detected in the root, stem, calyce, and petal of PP2OETAt1 (Figure 7d). GUS activity was not found in the flower stalk (Figure 7d), possibly due to no *uidA *expression or little GUS beyond detectable level. In addition, uidAETAt1 showed as tolerant as the transgenic control plant to phloem feeding by *M. persicae *(Table 4). This result indirectly suggests that *AtPP2-A1 *plays a role, only when expressed itself, in repressing the phloem-feeding activity.

**Table 4:** Analysis of major activities of aphid feeding from uidAETAt (*uidA*-expressing transgenic *Arabidopsis thaliana*) and transgenic control plants

Activity examined	Control plant (SD)	uidATEAt1 (SD)	Student's *t*-test (n = 20)
Total duration of nonpuncturing, min	23.5 (5.2)	21.9 (4.5)	*

Duration of pathway phase, min	181.8 (56.0)	182.6 (61.6)	*

Total duration of phloem phase, min	34.7 (5.5)	35.5 (5.6)	*

*Insignificant difference at *p *< 0.05.

## Discussion

Although harpin proteins and their functions as proteinaceous elicitors in eliciting plant defense responses have been found for decades [1-3], many aspects of the mechanisms that underlie harpin-induced defenses remain unclear. Important questions include, for example, how a harpin protein as an exogenous signal is perceived by plants and how the signal perception is connected to a transducer to trigger a cellular pathway. Great attentions have been paid to plant signal transduction in harpin-induced resistance to pathogens [3,6,30,31,37,44] and insect herbivores [2,4,8,34]. We have used HrpN_Ea_, the first-characterized [1] and well-studied harpin [2-7], as a model of proteinaceous elicitors to characterize induced resistance against insect herbivores [2,4,8,34], particularly the green peach aphid *M. persicae*, a generalist phloem-feeding insect [10]. It has been shown that the HrpN_Ea _treatment and *M. persicae *infestation have some overlapping effects on the induction of plant responses [4,25,30-34], especially the PBD mechanism that is suggested to involve the lectin-type phloem protein PP2 [23,24] as a component [14-16,25,26]. Although 30 members of the *PP2 *multigene family have been identified in Arabidopsis [23] and *AtPP2*-modified Arabidopsis mutants generated [27], little is known about biological effects, especially on resistance to insects, of the genes and mutants http://www.arabidopsis.org. The purpose of this study is to elucidate the function of *AtPP2-A1 *in resistance to *M. persicae *in Arabidopsis plants when treated with HrpN_Ea _and under the condition of *AtPP2-A1 *overexpression.

We show that the treatment of Arabidopsis with HrpN_Ea _induces a repression in *M. persicae *feeding from the plant phloem (Figure 1; Table 1) and colonization of plants by the insect (Figure 2). Based on the EPG patterns, applying HrpN_Ea _to WT Arabidopsis impedes aphids in stylet puncturing of the plant cell, en route to the vascular tissue while looking for the phloem, and, especially, in the phloem-feeding activity. So the HrpN_Ea _treatment is likely to induce changes in cell wall properties unfavorable to aphid feeding, but this notion remains to be examined. HrpN_Ea_-induced deterrent effect on the phloem-feeding activity has been found in the EPG data analyzed either by hour or based on the four-hour record as a whole (Table 1). The phloem-feeding activity could be reflected in the EPG by duration of the phloem phase composed of E1 and E2 salivations (Figure 1), which are essential for ingestion of the phloem sap [13,16,17,35]. Shortened duration of the phloem phase, in both E1 and E2 salivations (Table 1), suggests that the insect's effort in ingestion of the phloem sap is repressed under the HrpN_Ea _treatment condition compared with control. It is also pertinent to propose that the HrpN_Ea _treatment impacts the insect-plant interaction. In terms of the insect, E1 and E2 saliva are believed to prevent protein clogging inside the sieve element and prevent phloem proteins from clogging inside the capillary food canal [13,16], respectively. In the plant side, phloem protein plugging of the sieve element presumably serves as a physical barrier to aphid feeding from the phloem [26]. The lectin-type phloem protein PP2 [23,24] is supposed to play a role in plant response to the feeding stress [21,25,26].

Molecular and genetic evidence supports a role of Arabidopsis *PP2 *gene *AtPP2-A1 *in HrpN_Ea_-induced repression of *M. persicae *feeding from the plant phloem. RT-PCR analyses (Figure 3) suggest that *AtPP2-A1 *is the most HrpN_Ea_-responsive gene of 30 members of the *PP2 *multigene family [23]. PLACE Web Signal Scan [39] provides a clue to molecular basis of HrpN_Ea _response and the *AtPP2-A1 *induction as well. For example, the gene promoter contains three copies of the consensus GT-1 binding box GA/GA/TAAA/T (#S000508) [45]. This element is involved in the regulation of salicylic acid signaling [45,46], which otherwise can be activated by HrpN_Ea _treatment in Arabidopsis [3]. Moreover, previous studies have shown that HrpN_Ea_-induced resistance to *M. persicae *is regulated by the ethylene signaling pathway [4], which essentially involves perception of the ethylene signal by the receptor ETR1, the signal transduction to the integral membrane protein EIN2 [4], and the regulation of ethylene responsive factors (ERFs) [47,48]. The W-box TGACC/T (#S000457) present in the *AtPP2-A1 *promoter has been shown as required for wounding-induced activation of the *ERF3 *gene [47]. The ERF3 protein is a regulator of ethylene signaling [48], which otherwise is activated to regulate induced resistance to *M. persicae *in Arabidopsis plants responding to HrpN_Ea _treatment [4]. Thus, *AtPP2-A1 *is pertinently thought a part of the signaling pathway that is required for HrpN_Ea _response, at least during induction of the plant resistance to *M. persicae *[4]. A role of *AtPP2-A1 *in the induced resistance has been elucidated by evidence obtained from investigating ten mutants (Table 2) in comparison with the WT plant. The investigation demonstrates that deterrent effect of the HrpN_Ea _treatment on aphid feeding from the phloem requires a functional *AtPP2-A1 *gene in the plant (Figure 4). This notion is especially supported by the absence of HrpN_Ea_-induced repression of the phloem-feeding activity in the *atpp2-*a1/E/142 mutant (Figure 4; Table 3). This result offers a novel angle to further understanding on the PBD mechanism. Previously, this defensive mechanism was known as a result of plant responses to attacks by phloem-feeding insects [14,15,20-22] and other stresses, such as wounding [16,21,22,26]. Now, the PBD mechanism is known to occur as a result of plant response to HrpN_Ea_, a proteinaceous elicitor of plant defenses [1-7,49]. This notion, however, remains to be examined in regard to how *AtPP2-A1 *contributes to PBD in response to the HrpN_Ea _treatment.

The function of *AtPP2-A1 *in conferring repression of the phloem-feeding activity is further supported by evidence obtained from investigating PP2OETAt (*AtPP2-A1*-overexpression transgenic *A. thaliana*) plants (Figure 5). Levels of *AtPP2-A1 *expression are significantly greater in the 10 tested PP2OETAt lines than in the transgenic control plant, conforming to the experimental design for the gene overexpression. In the different PP2OETAt lines, durations of the phloem phase are decreased with increases in levels of *AtPP2-A1 *expression, suggesting that *AtPP2-A1 *overexpression confers a repression in the phloem-feeding activity of *M. persicae*. These observations also identify the PP2OETAt1 line as the greatest expresser of *AtPP2-A1 *and the greatest repressor of colonization and feeding by the insect. Reminiscently of cell-to-cell *PP2 *RNA movement in cucurbits [50] and distant phloem transport flowering signals [51], little amount of the *AtPP2-A1 *transcript in flower stalks (Figure 6) may result from organ-to-organ trafficking to fulfill the requirement for flower development. In the other organs, however, *AtPP2-A1 *expression is consistent with the repression of aphid feeding from the organs (Figure 6). The organ-unspecific feature of *AtPP2-A1 *expression and function is also suggested indirectly by investigating the transgenic plant uidAETAt1. In the plant, *uid*A expression under direction of the *AtPP2-A1 *promoter is found in various organs (Figure 7), but these organs do not have a repressive effect on aphid feeding (Table 4). This result indirectly supported that *AtPP2-A1 *plays a role, only when expressed itself, in repression of the phloem-feeding activity. Consistent to our observations on uidAETAt1, a previous study detected GUS activity in different organs of transgenic plants that expressed *uidA *under direction by the *AtPP2-A *promoter [23]. In the present study, both *uidA *transcript and GUS activity were detected in different organs of uidAETAt1 (Figure 7). Due to our failure in obtaining transgenic plants that had been designed to express *AtPP2-A1-uidA *under control by the *AtPP2-A1 *promoter, now we can not provide more convening evidence for coincident organ localization in *AtPP2-A1 *expression and aphid feeding repression. However, repression of the phloem-feeding activity seems a consistent attribute of the different PP2OETAt lines (Figure 5) and a consistent attribute of the different organs of PP2OETAt1 (Figure 6) as well, owing to *AtPP2-A1 *overexpression in both cases. In the case of PP2OETAt1, whenever the level of *AtPP2-A1 *expression is greater in an organ than in the others, aphid feeing from the organ incurs a stronger repression (Figure 6). These observations offer a convincing support for the function of *AtPP2-A1 *in conferring the plant resistance shown as a repression in phloem-feeding activity of the insect. The results also indicate a defensive significance of ubiquitous organ-unspecific expression of *PP2 *genes in plants demonstrated previously [23] and observed in this study (Figure 7).

The contribution of lectin-type phloem proteins, such as PP2, to the PBD mechanism is believed owing to their functions as a physical barrier that prevents insects from phloem feeding [26]. A preceding event is the formation of the PP1-PP2 complex, which, however, has been demonstrated only in cucurbits, whereas, other plant families do not have any PP1-like protein [52]. The role of PP1-PP2 aggregation in the clogging of sieve plates has been the matter of long standing debates that have not yet been solved, and still remains a hypothesis that is beyond elucidating scopes of the present study. Lectin-type phloem proteins take only a small proportion of phloem sap proteins that have potential of defensive significance in plants under attacks by phloem-feeding insects [21]. Thus, lectin-type phloem proteins are only one of different PBD components and are not likely to play an entire role in plant resistance against attacks by the insects [14-21]. Subtle differences in aphid population, the insect escape from leaf colonies, for example, between HrpN_Ea_-treated plants and control plants (Figure 2), between *atpp2-a1*/E/142 and WT (Figure 4), and between PP2OETAt and transgenic control plants (Figure 5), also imply components alternative to *AtPP2-A1 *in impacting aphid behaviors while colonizing the plants. Alternative defense components are further indicated by HrpN_Ea_-induced impediments to aphid feeding activities observed in the first-hour EPG monitoring (Table 1). However, we do not have evidence yet to show a proportion of *AtPP2-A1*'s contribution to resistance against *M. persicae *in Arabidopsis plants either when treated with HrpN_Ea _or under the condition of *AtPP2-A1 *overexpression.

Moreover, *AtPP2-A1 *is a member of the *PP2 *multigene family [23,27] and *atpp2-a1*/E/142 is one of *AtPP2 *mutation alleles in Arabidopsis [24]. The other *AtPP2 *genes and *AtPP2*-modified mutants seem not involved in HrpN_Ea_-induced repression of aphid feeding from the phloem (Figure 4). This result suggests that different members of the *PP2 *multigene family may have different functions in the plant. So far, AtPP2-A1 is the only phloem protein demonstrated as a lectin with the ability to bind N-acetylglucosamine oligomers, and recombinant AtPP2-A1 has been shown to affect weight gain in *M. persicae *nymphs in an artificial diet [24]. The induction of *AtPP2-A1 *may be an indirect effect of the HrpN_Ea _treatment, which is multifunctional, inducing plant growth enhancement [4], resistance to pathogens [3], insects [4] and drought stress [5], and resistance-associated cell death [1,6]. These multiple effects have been determined separately; and so whether they are simultaneous is unclear. It is also unclear if other PP2 genes affect plant defenses rather than resistance. In several species of angiosperms, including Arabidopsis, different *PP2 *genes are expressed in various organs during plant growth and development [23]. The ubiquitous organ-unspecific *PP2 *expression suggests that different *PP2 *genes may fulfill distinct functions at a special stage of plant growth and development. It is possible that a particular *PP2 *gene may have different functions depending on plant growth and development processes or depending on an immediate requirement for encountering with distinct challenges, such as attacks by insects and infection by pathogens. Studies to test this hypothesis represent an interesting avenue for further research.

## Conclusions

The HrpN_Ea _treatment has a deterrent effect on the phloem-feeding activity of *M. persicae *and the deterrent effect occurs in WT Arabidopsis rather than the *atpp2-a1*/E/142 mutant. The phloem-feeding activity can be also repressed as a result of *AtPP2-A1 *overexpression. Both sets of evidence support the conclusion that *AtPP2-A1 *plays a role in Arabidopsis resistance to the insect, particularly at the phloem-feeding stage. The accompanied change of aphid population in leaf colonies suggests that the function of *AtPP2-A1 *is related to colonization of the plant and may have a broader importance for the plant-insect interaction.

## Methods

### Plant growth and treatment

Arabidopsis genotypes used in this study included the ecotype Col-0, transgenic plants created in this study, and *AtPP2-A *sequence-indexed T-DNA insertion lines generated previously (Table 2). Both types of modified plants were created under the background of Col-0 and characterized as homozygous at the transgene and T-DNA insertion loci, respectively, before use in the experiments. Plants were grown in 9-cm pots, 1 plant/pot for the EPG monitoring and 5 plants/pot for other experiments, under 22°C and 250 μE/m^2^/s illumination [53]. A long day (16-h light/8-h dark) photoperiod was applied to plants for transformation and *AtPP2-A1 *expression in different organs, and short day (with 12-h light/12-h dark) was used in other experiments. Plants grown in short day were used at different stages of growth and development depending on experimental purposes. Transgenic plants were used in different experiments since the 35th day after planting. Thirty-day-old plants of the WT and mutants were treated with EVP and HrpN_Ea_, respectively. EVP and HrpN_Ea _were prepared [1,6] as 10 μg/ml aqueous solutions and were applied in the presence of surfactant Silwet-77 (0.02%) by spraying plant tops with a low-pressure atomizer. Treated plants were used at 5 dpt in monitoring of aphid behaviors, and were used at 0 and 24 hpt in determination of gene expression.

### Aphid culture

A single isolate of *M. persicae *was collected from the field-grown radish (*Raphanus sativus *L.) near Nanjing in China. A clone of apterous agamic females was obtained by acclimatization in WT Arabidopsis grown in the chamber (22°C; 250 μE/m^2^/s; short day). The colony was maintained in nursery WT Arabidopsis seedlings and was transferred to fresh plants every two weeks. Uniform ten-day-old aphids were used in this study and were transferred to experimental plants with a fine paintbrush.

### Aphid feeding behavior

Aphid feeding activities were observed by the EPG technique using the Giga Amplifier system (Laboratory of Entomology, Wageningen Agricultural University, Wageningen, The Netherlands; http://www.epgsystems.eu/systems.htm). Uniform ten-day-old aphids were placed on upper side of the top first expanded leaves of plants. For each genotype of the plant or each combination of a genotype and treatment (with EVP or HrpN_Ea_), 20 aphids placed on 20 plants were monitored in five repetitions of experiments. Immediately after aphids were placed on leaves, a 20-mm diameter gold wire was attached to the dorsal surface of each aphid's abdomen using silver conductive paint. The other end of the wire was connected to a four-channel Giga-4 direct current amplifier with four channels and 10^9^-Ω input resistance in an electrical circuit that is also connected to the plant via an electrode placed in the soil. The behavior of individual aphids was monitored for 4 hours. Voltage waveforms were digitized at 100 Hz with an A/D converter USB device. Waveform patterns were identified according to previously described categories [35]. Waveform recordings were dissected each 5 second with the EPG analysis software STYLET 2.5 installed in a computer connected to Giga-4 direct current amplifier.

### Plant colonization

Uniform ten-day-old aphids were placed on the lower sides of the top two expanded leaves of plants; 10 aphids per leaf. A total of 1,200 aphids were monitored in four repetitions of the experiments for each single recombination of a treatment and a plant genotype. In each experimental repetition, 300 aphids were placed on 30 leaves of 15 plants treated specifically. Aphid movement from leaf colonies was monitored for five days, and the number of aphids in a leaf colony was scored at intervals in 24 hours. Aphid reproduction was surveyed twice a day, and in each survey, newborn nymphs were counted. Reproduction rate was quantified as the ratio between total numbers of nymphs produced in five days and total numbers of aphid adults that stayed in leaf colonies during the same period. Nymphs produced in five days were also monitored; the number of nymphs that run away from leaf colonies was accounted.

### Determination of gene expression in plants

Total RNA was isolated from leaves of EVP-treated plants and HrpN_Ea_-treated WT plants, and was isolated from leaves, stems, flower stalks, calyces, and petals of transgenic plants. Gene expression was determined by northern blot hybridization [31] and RT-PCR or real-time RT-PCR [54] as described previously. Northern blots of leaf RNA samples were hybridized to a digoxigenin-labeled *AtPP2-A1 *probe prepared using the DIG Nucleic Acid Detection Kit [Roche Diagnostics (Shanghai) Trading Co., LTD]. An established quantitative method [55] was adopted in real-time RT-PCR using *ACTIN2 *and *EF1α *as reference genes [54,56]. Genes were amplified <26 cycles with a range of template concentration increases by 0.5 ng and from 0 to 3.0 ng in 25 μl reaction solutions to select desired doses. Reaction treatments, RT-PCR protocols, product cloning and sequencing verification were performed as described [5,6]. The 25 μl reaction mixture was composed of 1 μl first-strand cDNA diluted 1:10, 2.5 μM primer and 1×SYBR Premix Ex Taq (TaKaRa Biotech. Co., Ltd, Dalian, China). All reactions were performed in triplicate with null-template controls in which cDNA was absent. PCR cycling was: 95°C for 3 min, followed by 40 cycles of 30 sec at 95°C, 30 sec at 60°C and 30 sec at 72°C. Average expression levels of the genes were normalized to the null-template controls. Average level of the *AtPP2-A1 *transcript was quantified relative to *EF1α *and *ACTIN2*. The expression of *uidA *in different organs of the uidATEAt plant was determined by RT-PCR using the superscript II RNAse Hˉ Reverse Transcriptase (Invitrogen Biotech. Shanghai Trading Co., LTD). Primers and related information are provided in Table 5.

**Table 5:** Information on genes analyzed by reverse transcriptase-polymerase chain reaction in this study

Gene	Locus no.	Primers	Product size (bp)
*ACTIN2*	AT3G18780	5'-CCCCTGAGGAGCACCCAGTTCTA-3', 5'-CATACCCCTCGTAGATTGGCACAG-3'	219

*AtPP2-A1*	AT4G19840	5'-GCCTAACGGTAAGGAGAA-3', 5'-TTACTGTTTGGGACGAAT-3'	205

*AtPP2-A2*	AT4G19850	5'-TCAATTACATGGGCAGAGTCTCAA-3', 5'-TCTCCACCCACTTGTTCCTTTCTA-3'	401

*AtPP2-A3*	AT2G26820	5'-TGTGGTGGACGGAAGGTGCT-3', 5'-CCTCCTGGCCTACTGTTGATGTAAAA-3'	716

*AtPP2-A4*	AT1G33920	5'-GATCTACGCAAGGGATCTTAGCATT-3', 5'-CTCCAGCATTATCTGGTGATGTCACGAACT-3'	371

*AtPP2-A5*	AT1G65390	5'-GTAAAGTCAATCGTCAAGGCTGTTAA-3', 5'-TTCTCCCAAGTATTCGGCAAGTC-3'	524

*AtPP2-A6*	AT5G45080	5'-ATGGCTTCTTCTTCCTCGGTTGTG-3', 5'-GAGTTTGGTGCCTCGTTGATGGT-3'	797

*AtPP2-A7*	AT5G45090	5'-TAATGAATCCGCCGATGAAGC-3', 5'-CAACACCTTTGACCACGAGCC-3'	638

*AtPP2-A8*	AT5G45070	5'-AATGCGATTCCCATCTTCTACAAAC-3', 5'-CACTCATAACCACCTTCAGCGTCA-3'	565

*AtPP2-A9*	AT1G31200	5'-GTTCGCATCATAAGGCAGACTCCA-3', 5'-TTCTTGAACAAAGGCTTCGTGGA-3'	521

*AtPP2-A10*	AT1G10150	5'-AATCCCTAACAGCTTGAAGCAGATC-3', 5'-TGCAATAGCCTCAGTCCACCC-3'	694

*AtPP2-A11*	AT1G63090	5'-CGCTTCTTGGGCTGATTTCG-3', 5'-GACTCCAGTTTCCTGCTTCGGTTA-3'	533

*AtPP2-A12*	AT1G12710	5'-TTGTCTTCTTCATCTTGTTTTGGGG-3', 5'-CCGCTTCAACTGGTCTTTACACGAG-3'	837

*AtPP2-A13*	AT3G61060	5'-CAGATTGGTGGATTTACCTGAGAATT-3', 5'-TTGTTGGTTGTCCGAAGTGGC-3'	598

*AtPP2-A14*	AT5G52120	5'-AGACAAACTTATTTACCGC-3', 5'-AACTGCTTCTAACCACCAT-3'	244

*AtPP2-A15*	AT3G53000	5'-TTTCGTGGTGCGGCTTCTTC-3', 5'-TGCGTGCAGTCAATCTGTTTCAT-3'	659

*AtPP2-B1*	AT2G02230	5'-CGAGTCCTCGGGACGCTTGT-3', 5'-CCACGGACGCCTCATCCTAAA-3'	620

*AtPP2-B2*	AT2G02250	5'-CCGGTTCTTCGTCGATGGTG-3', 5'-AAGCCGAGTAACGGGTTCCAG-3'	537

*AtPP2-B3*	AT2G02270	5'-TTTTGCTGCTTCGGTTTCG-3', 5'-CCCATGAGATCACCATTCCCT-3'	792

*AtPP2-B4*	AT2G02280	5'-ATGAATACTCAAATCCTATC-3', 5'-TTATGGGCTTTTCGTAGGGCGGATA-3'	435

*AtPP2-B5*	AT2G02300	5'-GTTCCTTGCTGCTTTGGTTTCG-3', 5'-CCATCCACCCATCTTGCCTCT-3'	536

*AtPP2-B6*	AT2G02310	5'-TGGAATCTATCGGTGGAGGCG-3', 5'-CAACTTGTATAGGCAAATCTCGTAAGC-3'	570

*AtPP2-B7*	AT2G02320	5'-AGCCGTTGTCTTTGGGTGATTT-3', 5'-ACGTTTCGTATTGCGCTGAGTAG-3'	755

*AtPP2-B8*	AT2G02340	5'-TTCACAAGCCCTCAAGATGCG-3', 5'-CACCACTCCAACTACAACTTCTACGG-3'	498

*AtPP2-B9*	AT2G02350	5'-TGCAACTGCGATGAATCTATCAAG-3', 5'-CTGCTGGGCGTATTTACCCTCT-3'	448

*AtPP2-B10*	AT2G02360	5'-GCGTCGCTGCTACGGTTTCG-3', 5'-GCTCAATCTCCATCCACCCATCTT-3'	579

*AtPP2-B11*	AT1G80110	5'-TGCGGCACCTGCTGGTCTTC-3', 5'-CCCTTTGTCTCCTTGAGGCTCATCTC-3'	558

*AtPP2-B12*	AT5G24560	5'-GCGGCGGATTCCAATACCA-3', 5'-AAGTTCAATCTCCAACCACCCATC-3'	525

*AtPP2-B13*	AT1G56240	5'-CCAACATCCTTGCCTTCACATC-3', 5'-TCTCCAACCACCCGTCGTCT-3'	690

*AtPP2-B14*	AT1G56250	5'-ATAGCCAACATCCTTGCCTTCA-3', 5'-TCAATCTCCATCCATCCGTCAT-3'	698

*AtPP2-B15*	AT1G09155	5'-ATCTCGTCGGCGGCTGTCTC-3', 5'-CTATCTCCATCCACCCATCGTCTC-3'	649

*EF1α*	AT1G07930	5'-CCCCTTCGTCTCCCACTTCAGGATGTCTA-3', 5'-GTTGTCACCTGGAAGTGCCTCAAGAAG-3'	189

*Kan*^*r*^	HM047294	5'-GGCTATGACTGGGCACAACAGACAA-3', 5'-GCGGCGATACCGTAAAGCACGAGGA-3'	683

*uidA*	U00096	5'-GGGGTGGCAGTGAAGGGCGAACAGT-3', 5'-TGGGAGAACATTAGGTAGACGCAGGTGA-3'	533

### Mutant screening

Information on sequence-indexed T-DNA insertion Arabidopsis mutants tested in this study (Table 2) was from The Arabidopsis Information Resource (TAIR, http://www.arabidopsis.org) seed stock database. Mutant seeds were provided as either homologous (*atpp2-a1*/P/-210, *atpp2-a10*/P/-157, and *atpp2-a3*/I/1650) or heterozygous (the other seven mutants) at the insertion loci (Table 2). Homozygous progenies of heterozygous mutants were obtained by a PCR-based screening protocol according to information shown in Table 2. Provided mutant seeds were used to grow progeny plant lines and new seeds were harvested separately from five lines of each mutant. In the next generation, five lines of a mutant were grown for use to analyze the T-DNA insert and identify homozygous plants. Genomic DNA was isolated separately from ten plant individuals of each line and subjected to PCR analyses with *Kan*^*r*^-specific primers (Table 5). Seeds from the line that had *Kan*^*r *^in all the ten plant individuals were regarded as homozygous at the insertion locus.

### Promoter analyses

Promoter sequences of the *AtPP2-A *genes (Figure 3b) were predicted with the AtcisDB program http://arabidopsis.med.ohio-state.edu/. Presence and locations of plant *cis*-acting regulatory DNA elements in the promoter sequences were determined by analyses with the PLACE Web Signal Scan program http://www.dna.affrc.go.jp/PLACE/signalup.html[39]. The *cis*-acting regulatory DNA elements were correlated with genes and processes by browsing linked web information and publications.

### Generation and characterization of transgenic plants

The binary vector pBI121 (EMD Bioscience Inc., Gibbstown, NJ, USA), which contains the *NPT II *gene encoding kanamycin resistance, *35S *and *uidA*, was used to construct transformation units. Full length cDNA of the *AtPP2 *gene used in construction of pBI121::*35S*::*AtPP2-A1 *was obtained by RT-PCR conducted with RNA isolate from leaves of HrpN_Ea_-treated plants and *AtPP2-A1*-specific primers (5'-CGG*GATCC*ATGAGCAAGAAACATTGCTCAG-3' and 5'-CG*AGCTC*TTACTGTTTGGGACGAATTGCAACAC-3'; underline indicates protection bases; italics indicate *Bam*H I and *Sac *I restriction bases). The gene was inserted into the pBI121 vector at the *Bam*H I and *Sac *I restriction sites to replace *uidA *(Figure 5a). The AtPP2-A1 promoter was obtained by PCR using the genomic DNA from WT plant and the specific primers (5'-CC*CAAGCTTG*ATAATTTTTCAAGACCC-3' and 5'-CGG*GATCC*AAACCAGTATGATGTATT-3'; underline indicates protection bases; italics indicate *Hind *III and *Bam*H I restriction bases). The promoter sequence was inserted pBI121 at the *Hind *III and *Bam*H I restriction sites to replace *35S *(Figure 7a), creating pBI121::*PP2P*::*uidA*.

Recombinant vector was transferred into cells of *Agrobacterium tumefaciems *strain EHA105. A suspension of EHA105 cells containing the empty pBI121 vector (without *AtPP2-A1 *and *uidA *inserts) or the recombinant vector were used to transform WT Arabidopsis by blossom infiltration. Transformation with pBI121::*GUS*::*AtPP2-A1 *and pBI121::*PP2P*::*uidA *generated PP2OETAt and uidAETAt plants, respectively. Both types of transgenic plants were screened, multiplied and characterized by a previously described protocol [31]. The phenotype of kanamycin resistance was used in screening of PP2OETAt candidates and the transgenic control plant candidates, respectively; the phenotype of both kanamycin resistance and GUS activity was used in screening of uidAETAt candidates. GUS activity was determined using the histochemical techniques described previously [57]. Screened transgenic plants were allowed to self-pollinate and selected to the T3 generation [58]. T3 homozygous progenies were used in this study. The genomic integration of the transgene in PP2OETAt was detected by Southern blot analysis [54]. For Southern blots, Arabidopsis genomic DNA was digested with *Bam*H I and *Sac *I, and transferred to a nylon membrane, followed by hybridization to a digoxigenin-labeled *AtPP2-A1 *probe prepared using the DIG Nucleic Acid Detection Kit [Roche Diagnostics (Shanghai) Trading Co., LTD].

### Data treatment

Experiments were done three or four times with similar results. The student's *t*-test was used to compare data obtained from HrpN_Ea_-treated plants with those obtained from EVP-treated plants, and to compare data obtained from the transgenic control plant with those obtained from each line of PP2OETAt. Quantitative data were also analyzed by the *ANOVA *test to compare differences among the transgenic control plant and different lines of PP2OETAt, and among different organs of transgenic plants.

## Authors' contributions

CZ, XW and SZ carried out EPG studies, investigated insect population, and performed the statistical analysis. CZ and LC also participated in the design of the study and drafted the manuscript. HS and BL did bioinformatics analyses and determined gene expression. HS also investigated aphid nymphs. XW, LY and RL generated and characterized transgenic plants. BL, JQ, WS and ZY participated in the insect monitoring experiments. HD conceived of the study, and participated in its design and coordination and helped to draft the manuscript. All authors read and approved the final manuscript.
